# Clinical efficacy and safety of polymyxins based versus non-polymyxins based therapies in the infections caused by carbapenem-resistant *Acinetobacter baumannii*: a systematic review and meta-analysis

**DOI:** 10.1186/s12879-020-05026-2

**Published:** 2020-04-21

**Authors:** Cheng Lyu, Yuyi Zhang, Xiaofen Liu, Jufang Wu, Jing Zhang

**Affiliations:** 1 Institute of Antibiotics, Huashan Hospital, Fudan University, 12 Middle Wulumuqi Road, Shanghai, 200040 China; 2 Key Laboratory of Clinical Pharmacology of Antibiotics, National Health Commission, Shanghai, China; 3 Shanghai Public Health Clinical Center, Shanghai, China

**Keywords:** Polymyxin, Colistin, Carbapenem-resistant *Acinetobacter baumannii*, Meta-analysis

## Abstract

**Background:**

The prevalence of infections due to carbapenem-resistant *Acinetobacter baumannii* (CRAB) is on the rise worldwide. Polymyxins are considered as last-resort drugs for CRAB infections, but there is still controversy regarding the efficacy and safety of polymyxins based therapies in CRAB infections. The present systematic review was designed to compare the efficacy and safety of polymyxins based therapies versus non-polymyxins based therapies in CRAB infections.

**Methods:**

We performed a systematic literature search in PubMed, Embase, CINAHL, Cochrane Library, and clinicaltrials.gov to identify eligible studies reporting the clinical outcomes of patients with CRAB infections. The meta-analysis employed a random-effects model to estimate the odds ratio (OR) and standardized mean difference (SMD) with 95% confidence interval (CI). The primary outcome was 1-month mortality for any cause. We also examined clinical response, microbiological response, length of stay in hospital, and adverse events.

**Results:**

Eleven eligible studies were analyzed (1052 patients in total), including 2 randomized clinical trials. Serious risk of bias was found in 8 out of the 11 studies. There was no statistically significant difference between polymyxins based therapies and non-polymyxins based therapies in 1-month mortality for any cause (OR, 0.95; 95% CI, 0.59 to 1.53), microbiological response (OR, 3.83; 95% CI, 0.90 to 16.29) and length of stay in hospital (SMD, 0.24; 95% CI, − 0.08 to 0.56). The pooled OR of clinical response indicated a significant difference in favor of polymyxin based therapies (OR, 1.99; 95% CI, 1.31 to 3.03). The pooled OR of adverse events showed that non-polymyxins based therapies were associated with fewer adverse events (OR, 4.32; 95% CI, 1.39 to 13.48).

**Conclusion:**

The performance of polymyxins based therapies was better than non-polymyxin based therapies in clinical response rate and similar to non-polymyxin based therapies in terms of 1-month mortality and microbiological response in treating CRAB infections. Due to the limitations of our study, we cannot draw a firm conclusion on the optimal treatment of CRAB infections, but polymyxins would be a relatively effective treatment for CRAB infections. Adequate and well-designed large scale randomized controlled trials are required to clarify the relative efficacy of polymyxins based and non-polymyxins based therapies.

## Background

*Acinetobacter baumannii* is a gram-negative opportunistic pathogen [1] and a member of the ESKAPE (*Enterococcus faecium*, *Staphylococcus aureus*, *Klebsiella pneumoniae*, *Acinetobacter baumannii*, *Pseudomonas aeruginosa*, and *Enterobacter*) pathogens. *A. baumannii* can develop diverse mechanisms of resistance and so is capable of escaping from the effects of the commonly used antibiotics [2]. It may cause a range of nosocomial infections, including pneumonia, bacteremia, wound infection, and post-neurosurgical meningitis, threatening the lives of patients, particularly in the setting of intensive care unit (ICU) [3].

Carbapenems are considered as the first-line agents for treating *A. baumannii* infections if the isolates are susceptible. But the widespread use of carbapenems since 1990 has provoked the emergence of carbapenem-resistant *A. baumannii* (CRAB) [4]. Lob et al. reported that only about 8–26% *A. baumannii* isolates were susceptible to carbapenems worldwide [5]. Alarmingly, the prevalence of CRAB isolates increased from 13.3% in 2004 to 70.5% in 2014 in China [6].

Polymyxins include polymyxin B and polymyxin E (also known as colistin). They were discovered in 1947 [7] but discontinued shortly thereafter due to high nephrotoxicity [8]. In recent years, they are reintroduced into clinical practice for the activity against many multidrug-resistant gram-negative bacilli. Polymyxins may provide a synergistic effect with other antibiotic classes by disrupting the outer membrane of gram-negative bacteria [9].

Currently, polymyxins are the most commonly used agents and often considered as the “last resort” or salvage treatments of CRAB infections [10]. However, there are still many controversies and confusions regarding the efficacy and safety of polymyxins based therapies in treating CRAB infections. While numerous reports showed good therapeutic effects of polymyxins based therapies [11, 12], some reports linked polymyxins based therapies with a higher mortality [13, 14]. Other antibiotics, such as tigecycline and sulbactam, alternative therapeutic options against CRAB, also have shown mixed clinical outcomes [15]. Therefore, it is important to elucidate whether the polymyxins based therapies are more effective than other alternative treatments in patients with CRAB infection.

This meta-analysis was designed to evaluate the efficacy and safety of polymyxins based therapies versus non-polymyxins based therapies in CRAB infections based on all the evidence available in the literature.

## Methods

The study design of this systematic review and meta-analysis was consistent with the Preferred Reporting Items for Systematic Reviews and Meta-Analysis Protocols (PRISMA-P 2015) Guidelines [16].

### Search strategies

Databases including PubMed, Embase, CINAHL, Cochrane Library and clinicaltrials.gov (www.clinicaltrials.gov) were searched for all the studies reporting the treatment of infections caused by CRAB from their inception to October 2019. We used the following search string: “polymyxin or colistin” and “*Acinetobacter baumannii* and drug-resistant” or “*Acinetobacter baumannii* and carbapenem” or “carbapenem-resistant *Acinetobacter baumannii*” (see Additional file 1 for detailed search strategy). Furthermore, references listed in the identified articles and other reviews were also searched to select relevant studies. No restrictions of language, publication year or publication status were applied.

### Selection criteria

Studies for inclusion were based on the following criteria: (1) adult patients with CRAB infection, (2) polymyxins group was polymyxins based therapy and control group was non-polymyxins based therapy, (3) randomized controlled trials (RCTs) or cohort studies or case-control studies (whether retrospective or prospective), (4) at least one active antibiotic was used in treatment groups, and CRAB isolates were sensitive to polymyxins in the polymyxins group, (5) carbapenem resistance was clearly defined as resistant to imipenem and/or meropenem (without limitation on definition of cut off value), (6) 1-month mortality for any cause was reported. Studies for exclusion were based on the following criteria: (1) animals, in vitro, pharmacokinetics/pharmacodynamics (PK/PD) studies or single-arm studies, (2) polymyxins were used in all treatment groups, (3) studies in patients infected with mixed microorganisms, (4) abstracts presented at conferences, editorials, reviews, systematic reviews, and meta-analyses, (5) less than five cases per treatment group were reported. We attempted to contact the authors for details if the data were unclear or missing.

Full-text articles were retrieved for the studies that fulfilled selection criteria. Two authors (CL & YZ) further checked the eligibility of each study independently.

### Outcomes

In this study, the primary outcome was 1-month (28–30 days) mortality for any cause (1-month mortality). The secondary outcomes were clinical response, microbiological response, length of stay in hospital and adverse events. Clinical response was defined as complete resolution of at least two signs of infection (such as abnormal temperature, leukocytosis or leukopenia) at the end of treatment. The signs of infection varied due to the site of infection. Clinical judgment was made by the clinician according to local guidelines. The microbiological response was defined as a negative microbiological culture, which was obtained at the end of treatment. Length of stay in hospital was measured from the date of the infection was diagnosed to the date of discharge or death. Adverse events included nephrotoxicity, hepatotoxicity, skin rash, and diarrhea.

### Data extraction and quality assessment

The information of first authors, publication years, study years, countries, study designs, patient demographics (including age, gender, resistance profile of the bacteria, disease, and APACHE II score), clinical settings, sample sizes, interventions (including regimen and route of administration) and clinical outcomes were extracted from individual studies. All data extraction was done independently and checked by two authors (CL and YZ) using a pre-defined data extraction form and then compared for verification. The risk of bias of individual studies was assessed by four authors (CL, YZ, XL & JW) using the Risk of Bias in Non-randomized Studies of Interventions (ROBINS-I) tool endorsed by Cochrane Scientific Committee for observational studies [17]. We assessed the risk of bias for seven domains including bias due to confounding, selection of participants into the study, classification of the intervention, deviations from intended interventions, bias due to missing data, measurement of outcomes, and selection of the reported result. The rating of each domain ranged from low, moderate, serious, to critical risk, and no information (NI). The rating of risk of bias was based on the data we used for meta-analysis rather than the data in publications. Any discrepancies were settled by consensus.

### Statistical analysis

We performed a meta-analysis using the Mantel-Haenszel method (random-effects model) and inverse variance approach (random-effects model) to estimate the Odd Ratio (OR) and standardized mean difference (SMD) with 95% confidence interval (CI) to compare polymyxins based therapies with non-polymyxins based therapies. Dichotomous variables including 1-month mortality, clinical response, microbiological response, and adverse events were described by OR and CI. Continuous variable, length of stay in hospital, was described by SMD and CI. Heterogeneity between studies was assessed by the Q statistics and *I*^2^ tests (*P* < 0.05 and *I*^2^ > 50% suggesting significant heterogeneity). Sensitivity analyses were performed on efficacy outcomes to identify the source of heterogeneity by excluding the studies with serious risk of bias, excluding studies published before 2010, excluding studies with small sample size and excluding studies with inadequate balance in baseline characteristics. The leave-one-out analysis was conducted to ensure that no single study unduly influenced the overall effect size. A funnel plot was used to measure the publication bias. Egger’s test and Peters’ test were used to evaluate the asymmetry of funnel plot [18]. Subgroup analyses were done to compare the efficacy split by infection site, route of administration and region. All the above analyses were performed with Review Manager (RevMan) software, Version 5.3, (Copenhagen: The Nordic Cochrane Centre, The Cochrane Collaboration, 2014.) except the analysis of publication bias, which was done with STATA software, Version 13.0. (StataCorp LLC, 2013).

## Results

### The selection of included studies

**Fig. 1:**
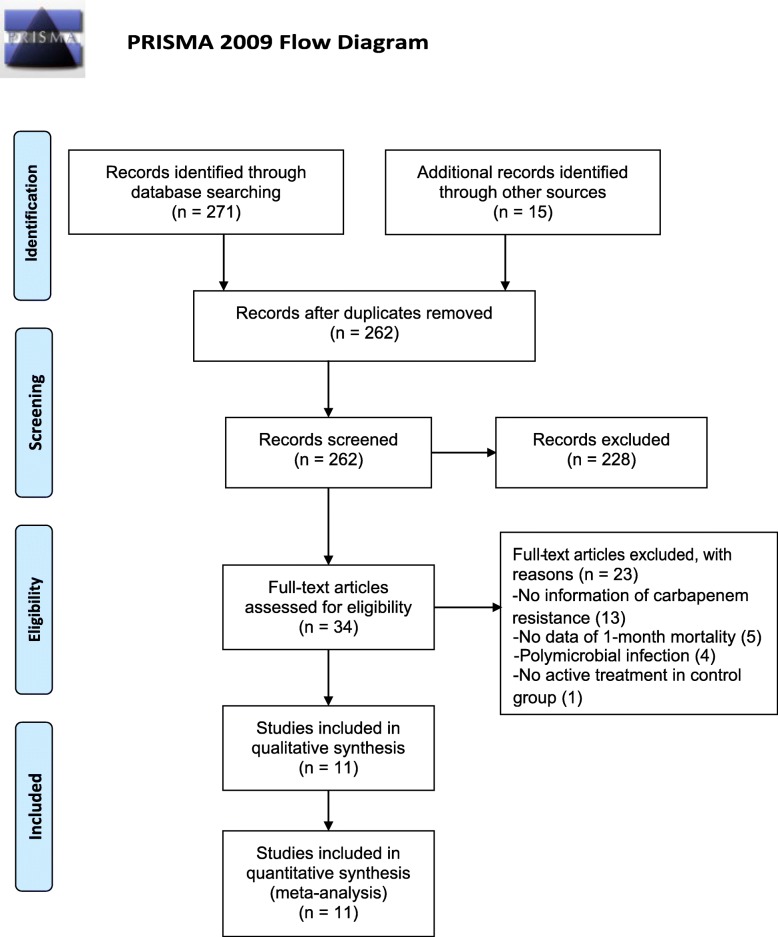
A PRISMA flow chart of study selection

A total of 271 studies were identified through the electronic database search and 15 studies were identified through manual search. After removing the duplicate records between databases, the abstracts of the remaining 262 articles were retrieved for a preliminary screening. Thirty-four studies with full texts were further assessed for eligibility, of which 13 studies were excluded because of insufficient information of carbapenem-resistance, 5 were excluded due to the unavailability of the data of 1-month mortality, 4 were excluded because patients were co-infected with other bacteria and 1 study was excluded because patients in control group did not receive active treatments. Finally, a total of 11 studies were included in our meta-analysis. Additional data were obtained from the author of the paper published by Raz-Pasteur and colleagues in 2019 [19]. The data were adequate for comparing colistin with ampicillin-sulbactam and comparing colistin with trimethoprim-sulfamethoxazole separately in two independent sets of data. Therefore, a total of 12 datasets were analyzed. A flowchart of study selection is shown in Fig. 1.

### Study characteristics

The meta-analysis was performed with 11 studies [13, 14, 19–27], including a total of 1052 patients (496 patients in polymyxins group and 556 patients in control group). The characteristics of the included studies are listed in Table 1. These 11 studies consisted of 8 retrospective studies [13, 14, 19, 22, 24–27], 2 RCTs [20, 21] and 1 prospective study [23]. The research hospitals are in North America and Europe (USA, Greece, Spain, Turkey, and France), Middle East (Iran, and Israel) and Asia (Taiwan, China, and Thailand). Two studies [25, 26] assessed patients with intracranial infections (such as meningitis). One study [13] reported on patients with intra-abdominal infection. Five studies examined pneumonia [14, 20–22, 24], including hospital-acquired pneumonia (HAP) and ventilator-associated pneumonia (VAP). Three studies [19, 23, 27] included more than one site of infection. In polymyxins group, colistin was administered in 10 studies [13, 14, 19–24, 26, 27] and polymyxin B [25] was used in only one study. In the control group, carbapenems, tetracyclines, cephalosporins, β-lactam/β-lactamase inhibitors, quinolones, aminoglycosides and glycylcycline (tigecycline) were the most commonly used alternative antibiotics. In 7 studies [13, 14, 19–21, 23, 24], the intravenous (IV) route of administration was used in both polymyxins and control groups. But in the other 4 studies [22, 25–27], patients in polymyxins group received IV and intrathecal/intracerebral (IT) polymyxins or IV and inhaled (IH) polymyxins, while patients in control group were given IV antibiotics only. One-month mortality was reported in all studies, clinical and microbiological response was reported in 5 studies. The length of stay in hospital and adverse events were reported in 4 studies.

**Table 1:** Characteristics of included studies

Author; Year	Country	Study years	Study design	Setting	CRs MIC /mg/L	Type of infection	Male/ Female	Age (years)	Treatment regimen		Sample size (PXs vs. Non-PXs)	Route of colistin	APACHE II score (mean)
									Polymyxins group	Non-polymyxins group			
Trottier 2007 [27]	USA	2004–05	RS	MIX	NA	Mix^b^	83/33	17 ~ 90	COL	AMK; FEP; CPF; DOX; IMP; MNO; TZP	96 vs. 20	IV/IH/IV+ IH/IV + IT	NR
Betrosian 2008 [20]	Greece	NA	RCT	ICU	NA	VAP	14/14	adult	COL	SAM	15 vs. 13	IV	14
Lopez-Cortes 2014 [23]	Spain	2010	PS	NA	≥32	RT/SS/SST UT/IAI/CNS OA/Bm	NA	>18	COL; COL+TGC; COL+CRs; COL+SUL; COL+AG; COL+RIF; COL+TGC + AG; COL+TGC+ CRs + AG	CRs; TGC; SUL; TCY; CRs + TGC; CRs + AG; TGC + RIF; TGC + AG; TGC + CRs + RIF	52 vs. 12	IV	NA
Ozvatan 2016 [24]	Turkey	1996–2010	RS	NA	NA	VAP	NA	≥18	COL; COL+OTH	M/I; SAM; CSL; OTH	29 vs. 187	IV	NA
Zalts 2016 [14]	Israel	2008–09	RS	ICU	NA	VAP	70/28	>18	COL	SAM	66 vs. 32	IV	17.5
Pan 2018 [25]	China	2013–17	RCS	NA	NA	ICI	30/31	>18	PB+ CTR	M/I + AMK; M/I + TGC; M/I + CSL; M/I + TGC + CSL; CSL; CSL + AMK;	23 vs. 38	IV + IT	18
Khalili 2018 [21]	Iran	2015–17	RCT	ICU	≥0.08	VAP	35/12	18 ~ 75	MEM + COL	MEM + SAM	24 vs.23	IV	NA
Liang 2018 [22]	Taiwan	2010–15	RS	MIX	NA	Pmn	167/71	>20	TGC + COL	TGC; TGC + OTH; SUL; SUL + OTH	110 vs. 128	IV/IV+ IH	23
Sipahi 2018 [26]	Turkey & France	2007–16	RS	ICU	NA	Meg	12/8	>18	TGC + COL	TGC + NET; TGC + AMK; TGC + MEM; TGC	8 vs. 12	IV/ IV + IT	NA
Chusri 2019 [13]	Thailand	2012–17	RS	MIX	≥16	IAI	16/12	>18	TGC + COL	TGC	14 vs. 14	IV	15/median
Raz-Pasteur 2019 [19]	Israel	2013–15	RCS	MIX	NA	Pmn/SST/Bm	62/21 72/40	Any	COL	SAM; TMP-SMX	59 vs. 24 59 vs. 53	IV	NA

*AG* aminoglycosides, *AMK* amikacin, *Bm* bacteremia, *CNS* central nervous system, *COL* colistin, *CPF* ciprofloxacin, *CRs* carbapenems, *CSL* cefoperazone-sulbactam, *CTR* ceftriaxone, *DOX* doxycycline, *FEP* cefepime, *FQs* fluoroquinolones, *IAI* intra-abdominal infection, *ICI* intracranial infection, *ICU* intensive care unit, *IH* inhaled, *IMP* imipenem, *IT* intrathecal/intracerebral, *IV* intravenous, *Meg* meningitis, *MEM* meropenem, *M/I* meropenem/imipenem, *MIC* minimum inhibitory concentration, *MIX* intensive care unit and hospital, *MNO* minocycline, *NA* not available, *NET* netilmicin, *OA* osteoarticular, *OTH* other antibiotics, *PB* polymyxin B, *PCS* prospective cohort study, *Pmn* pneumonia, *PS* prospective study, *PXs* polymyxins, *RCS* retrospective cohort study, *RCT* random clinical trial, *RIF* rifampicin, *RS* retrospective study, *RT* respiratory tract, *SAM* ampicillin-sulbactam, *SST* skin and soft tissue, *SUL* sulbactam, *TCY* tetracycline, *TGC* tigecycline, *TMP-SMX* trimethoprim-sulfamethoxazole, *TZP* piperacillin-tazobactam, *UT* urinary tract, *VAP* ventilator-associated pneumonia, vs. versus

### Quality assessment

**Fig. 2:**
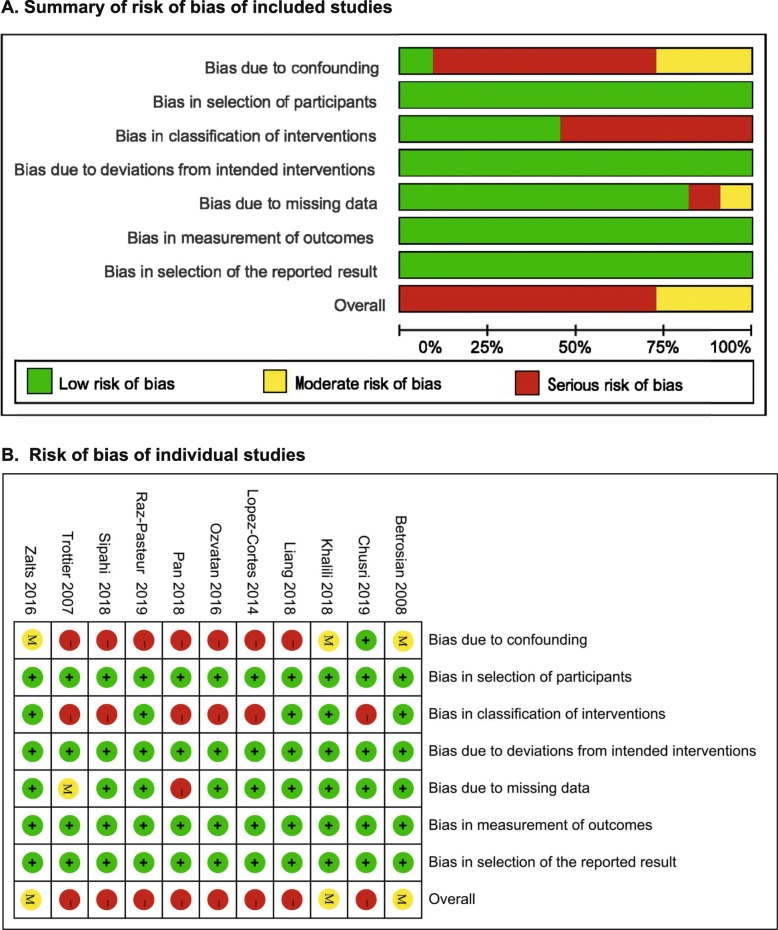
Summary of risk of bias: **a** Summary of risk of bias of included studies; **b** Risk of bias in individual studies. The green, yellow and red represent “low risk of bias”, “moderate risk of bias” and “serious risk of bias”, respectively

The output of the ROBINS-I tool is summarized in Fig. 2. The risk of bias was rated as moderate for 2 RCTs [20,21] and one [14] retrospective study, serious for the remaining 8 studies. Seven studies [19, 22–27] were at serious risk of bias in the domain of “bias due to confounding”. The common confounding factors were comorbidities (especially immunodeficiency), mixed infection sites, antibiotic susceptibility, severity of disease, and empirical treatment. Six studies [13, 23–27] were judged to be at serious risk in the domain of “bias in classification of interventions” because of lacking records of the dose/loading dose, frequency, and timing of intervention. Only one study was at serious risk of bias due to missing 50 records of patients without explanation [25].

### Meta-analysis for 1-month mortality

**Fig. 3:**
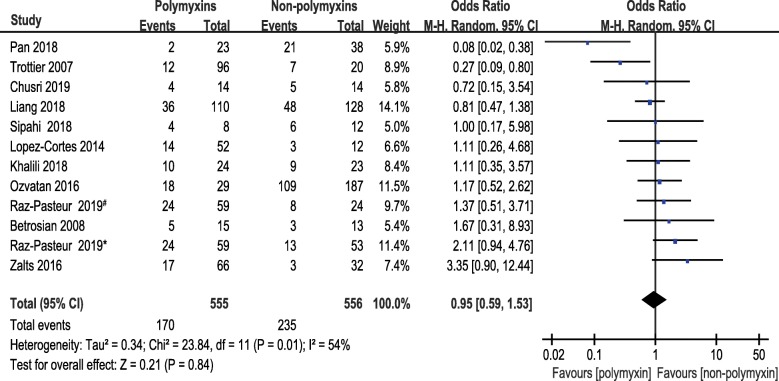
The forest plot of studies reporting 1-month mortality. Studies were ranked according to their effect sizes. #Ampicillin–sulbactam was used in non-polymyxins group. *Trimethoprim-sulfamethoxazole was used in non-polymyxins group

Eleven studies reporting the 1-month mortality of polymyxins based therapies and non-polymyxins based therapies for CRAB infections were included in this meta-analysis. There was no statistically significant difference between the two groups in 1-month mortality (OR, 0.95; 95% CI, 0.59 to 1.53; *P* = 0.84). Statistically significant heterogeneity (*P* = 0.01, *I*^2^ = 54%) was observed (Fig. 3).

### Meta-analysis for clinical response

**Fig. 4:**
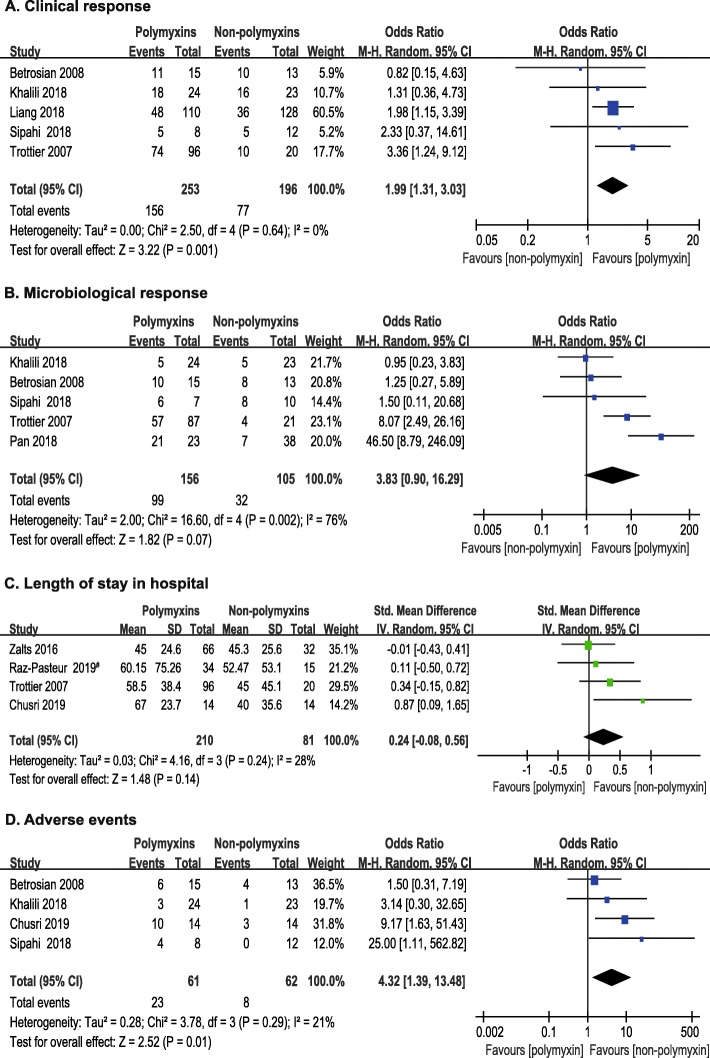
The forest plots of secondary outcomes: **a** clinical response; **b** microbiological response; **c** Length of stay in hospital; **d** Adverse events. Studies were ranked according to their effect sizes. #Ampicillin–sulbactam was used in non-polymyxins group

Five studies [20–22, 26, 27] involving 449 patients compared the clinical response of two types of therapies. No statistically significant heterogeneity was observed among these studies (*P* = 0.64, *I*^*2*^ = 0.0%). The pooled OR of clinical response suggested that polymyxins based therapies may have an advantage over non-polymyxins based therapies (OR, 1.99; 95% CI, 1.31 to 3.03; *P* = 0.001) (Fig. 4a).

### Meta-analysis for microbiological response

Five studies [20, 21, 25–27] (261 patients) reported the microbiological response. The microbiological response rates favored polymyxins group, but the difference was not statistically significant (OR, 3.83; 95% CI, 0.90 to 16.29; *P* = 0.07) between the two types of therapies. Statistically significant heterogeneity (*P* = 0.002, *I*^*2*^ = 76%) was observed among the five studies (Fig. 4b).

### Meta-analysis for length of stay in hospital

Four studies [13, 14, 19, 27] reported the length of stay in hospital. The heterogeneity observed among 4 studies was not statistically significant (*P* = 0.24, *I*^*2*^ = 28%). The length of stay in hospital did not differ significantly (SMD, 0.24; 95% CI, − 0.08 to 0.56; *P* = 0.14) between polymyxins based therapies (210 patients) and non-polymyxins based therapies (81 patients), but with a trend of prolonged hospitalization for patients receiving polymyxins based therapies (Fig. 4c).

### Meta-analysis for adverse events

Adverse events were recorded in 4 studies [13, 20, 21, 26] involving 123 patients. The main adverse events of polymyxins based therapies were associated with nephrotoxicity. Twenty nephrotoxicity-related adverse events were seen in the 61 patients with polymyxins based therapies, and 6 in the 62 patients treated with non-polymyxins based therapies. Our result showed that more adverse events occurred in polymyxins group than in control group (OR, 4.32; 95% CI, 1.39 to 13.48; *P* = 0.01) (Fig. 4d). Statistical heterogeneity was not significant among these studies (*P* = 0.29, *I*^*2*^ = 21%).

### Sensitivity analyses and publication bias analysis

We performed four sensitivity analyses to identify the source of heterogeneity by subgrouping. The planned exclusion was removal of studies with serious risk of bias, removal of studies published before 2010, removal of studies with small sample size, and removal of studies with inadequate balance in the baseline characteristics. The primary outcome and the two secondary outcomes (clinical response and adverse events) did not change substantially after subgrouping, which proved the consistency of our results (Additional file 2: Table S1).

A leave-one-out analysis was also performed to reflect the effect of individual dataset on the pooled ORs. The corresponding pooled ORs of our primary outcome were not changed remarkably (Additional file 2: Table S2).

The funnel plot was constructed to assess the publication bias of the literature (Additional file 3). There was no evidence of publication bias based on Egger’s test (*t* = − 1.21; *P* = 0.253) and Peters’ test (*t* = − 0.18; *P* = 0.864). The tests could be underpowered due to small sample size.

### Subgroup analyses

**Fig. 5:**
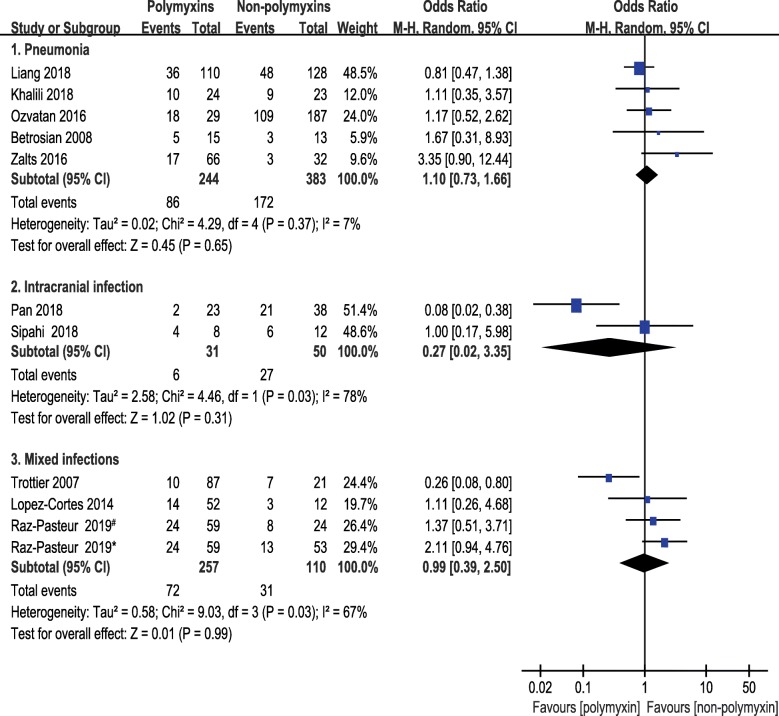
The forest plot of studies reporting 1-month mortality grouped by infection site. Studies were ranked according to their effect sizes. #Ampicillin–sulbactam was used in non-polymyxins group. *Trimethoprim-sulfamethoxazole was used in non-polymyxins group

**Fig. 6:**
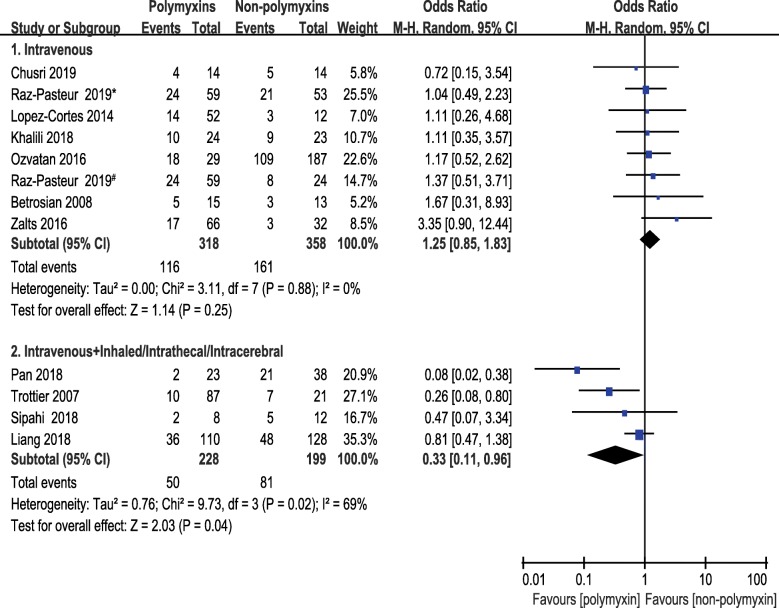
The forest plot of studies reporting 1-month mortality grouped by route of administration. Studies were ranked according to their effect sizes. # Ampicillin–sulbactam was used in non-polymyxins group. *Trimethoprim-sulfamethoxazole was used in non-polymyxins group

**Fig. 7:**
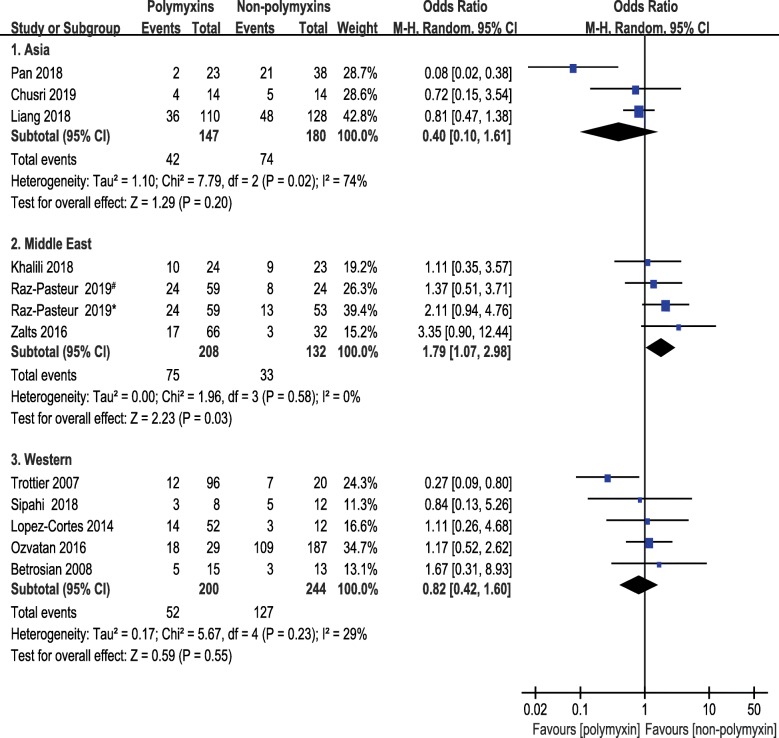
The forest plot of studies reporting 1-month mortality grouped by geographic region. Studies were ranked according to their effect sizes. #Ampicillin–sulbactam was used in non-polymyxins group. *Trimethoprim-sulfamethoxazole was used in non-polymyxins group

Subgroup analyses of 1-month mortality were planned to split the studies according to infection site, route of administration or region. Studies were classified into 4 subgroups in terms of infection site, i.e., pneumonia, mixed infection, intracranial infection, and intra-abdominal infection. The pooled ORs of 1-month mortality did not differ from the primary analysis remarkably. No significant difference was seen between polymyxins based therapies and non-polymyxins based therapies after subgrouping. The pooled ORs of pneumonia, intracranial infection and mixed infection were 1.10 (95% CI, 0.73 to 1.66), 0.27 (95% CI, 0.02 to 3.35) and 0.99 (95% CI, 0.39 to 2.50), respectively. Only one paper reported the intra-abdominal infection caused by CRAB and the OR was 0.72 with 95% CI (0.15 to 3.54) (Fig. 5). A subgroup analysis was carried out based on route of administration to compare the 1-month mortality between a subset of studies in which antibiotics were given intravenously in both groups and a subset of studies in which polymyxins were given by multiple routes. The pooled OR of studies, in which multiple routes (IV plus IH/IT) were used for polymyxins administration, was 0.33 (95% CI, 0.11 to 0.96). The pooled OR was 1.25 (95% CI, 0.85 to 1.83) in studies that antibiotics were administered intravenously only (Fig. 6). This result indicated that lower mortality was associated with the direct delivery of polymyxins to the focus of infection by intrathecal/intracerebral ventricle injection or inhalation. A subgroup analysis was also carried out according to geographical region in order to understand the regional difference of 1-month mortality. Interestingly, the result showed that studies from Middle East region were in favor of non-polymyxin therapies because of lower mortality rate (OR, 1.79; 95% CI, 1.07 to 2.98) (Fig. 7).

## Discussion

Infections due to CRAB require special attention. The high morbidity/mortality, the potential to cause outbreaks and the spread of antibiotic resistance are all associated with CRAB infections [3]. Polymyxins are the common options for CRAB infections in clinical practice nowadays. Are polymyxins actually the optimal choice for treating CRAB infections? We aim to answer this question by compiling the up-to-date knowledge on the efficacy and safety of polymyxin-based and non-polymyxin-base therapies for CRAB infections.

The goal of all medical treatments is to improve the overall survival of patients. The all-cause mortality is a measurement of overall survival and an objective endpoint which is not subject to bias. That is why we chose 1-month mortality for any cause as the primary efficacy outcome in this meta-analysis. And one-month follow-up period is commonly used in hospitals [28]. One-month mortality for any cause was 30.6% in polymyxins group and 42.3% in non-polymyxins group. Our meta-analysis showed that polymyxins based therapies were not associated with lower 1-month mortality when comparing with non-polymyxins based therapies. Many uncertainties are related to 1-month mortality. Especially in our study, 193 patients were in ICU settings with serious comorbidities and complications. Many unknown and undocumented factors could influence the outcome of 1-month mortality.

To further comprehend the relative efficacy on a more specific level, clinical and microbiological responses were designed as secondary outcomes. Our meta-analysis demonstrated that the performance of polymyxins based therapies (desirable clinical response in 61.7% patients) was better than non-polymyxins based therapies (desirable clinical response in 39.3% patients) in terms of clinical response. The percentage of patients achieving a good microbiological response at the end of treatment was 63.5% in polymyxins group, which was 2 times higher than that in non-polymyxins based group (30.5%). The microbiological response rates favored polymyxins group, but the statistical significance was not reached, probably due to small sample size.

Just like previous reports [29], we found that more adverse events were reported in polymyxins group, especially nephrotoxic events. A total of 23 adverse events were recorded in polymyxins group, of which 20 were associated with nephrotoxicity. We cannot emphasize enough the importance of renal function monitoring and timely dose adjustment for patients receiving polymyxins. The key was to follow the guidelines of polymyxins usage to balance the efficacy and safety [30, 31].

The subgroup analysis based on route of administration showed that direct delivery of polymyxins to the focus of infection helps. This was in agreement with the previous studies reporting the efficacy of IV polymyxins combined with inhaled or intracranial polymyxins, which was significantly superior to IV polymyxins alone for treating CRAB induced pneumonia and meningitis [32–34]. Limited penetration into alveoli and limited ability to cross the blood-brain barrier of polymyxins can partly explain our findings [35, 36].

A previous meta-analysis by Liu et al. in 2014 noted that polymyxins may be effective for treatment of *A. baumannii* infection [37]. However, the cases included in their analysis did not share a clear definition of carbapenem resistance. They evaluated the treatments of *A. baumannii* infections showing different types of drug resistance or no resistance. Given the importance of distinguishing drug resistance in antimicrobial treatments, a clear definition of drug resistance in our study selection could reduce the heterogeneity of meta-analysis. Furthermore, only the studies involving adult patients were included in our analysis, while Liu et al. compiled studies of both adult and pediatric patients. Considering the unique physiological and microbiological conditions of pediatric patients, antimicrobial therapy targeting resistant microorganisms should be discussed in pediatric patients separately. This report is therefore a more robust meta-analysis with improved study design.

Several limitations exist in our meta-analysis. First, the quality of the included studies was low. Only 3 of the 11 studies were at moderate risk of bias and the others were at serious risk of bias. Second, the studied population was heterogeneous. The diverse baseline conditions of infections and comorbidities introduced unknown confounding factors. Third, lacking information about drug use for comorbidity may cause the underestimation of unknown drug-drug interaction which affected the clinical outcomes. Furthermore, the sample size was small which could reduce the power of our analysis. Taking together, the findings must be interpreted with caution. Our results may be not representative enough for the whole picture of the polymyxins based and non-polymyxins based therapies against CRAB infections.

## Conclusion

Although our study cannot provide an answer for the optimal treatment of CRAB infection, our results did show that polymyxin could be a relatively effective therapy for CRAB infection. More randomized trials are needed to address the exact efficacy of different anti-CRAB treatments. Renal function monitoring to adjust the dose of polymyxin accordingly could largely improve the safety of polymyxin treatment [31]. Stewardship of polymyxin use also helps to prevent the emergence of polymyxin-resistant bacteria. More importantly, all healthcare providers should work closely to keep prioritizing research and development of new antibiotics.

## Supplementary information

**Additional file 1.** Search Strategies.

**Additional file 2: Table S1**. Summary of sensitivity analyses. Table S2. Summary of leave-one-out analysis on primary outcome.

**Additional file 3.** Funnel plot showing publication bias of 1-month mortality.

## Data Availability

The datasets used and/or analyzed during the current study are available from the corresponding author on reasonable request.

## Abbreviations

CI: Confidence interval
CRAB: Carbapenem-resistant *Acinetobacter baumannii*
ESKAPE: *Enterococcus faecium, Staphylococcus aureus, Klebsiella pneumoniae, Acinetobacter baumannii, Pseudomonas aeruginosa,* and *Enterobacter spp*
HAP: Hospital-acquired pneumonia
ICU: Intensive care unit
IH: Inhaled
IT: Intrathecal/intracerebral
IV: Intravenous
NI: No information
OR: Odds ratio
PK/PD: Pharmacokinetics/pharmacodynamics
RCT: Randomized controlled trial
ROBINS-I: Risk of Bias In Non-randomized Studies of Interventions
SMD: Standardized mean difference
VAP: Ventilator-associated pneumonia
