# Supplementary material for: Clinical efficacy and safety of polymyxins based versus non-polymyxins based therapies in the infections caused by carbapenem-resistant Acinetobacter baumannii: a systematic review and meta-analysis
Source: BMC Infect Dis. 2020 Apr 21;20:296. doi: 10.1186/s12879-020-05026-2 (PMC7175513; doi:10.1186/s12879-020-05026-2)
Supplement: Supplementary file 1 — Additional file 1. Search Strategies. [file 12879_2020_5026_MOESM1_ESM.docx]

**Search Strategies**

**PubMed**

| **No.** | **Query** |
| --- | --- |
| #5 | Search (((("Drug Resistance, Bacterial"[Mesh]) AND (("Acinetobacter baumannii") OR "Acinetobacter baumannii"[Majr]))) AND ((((((((( "Polymyxins/adverse effects"[Mesh] OR "Polymyxins/therapeutic use"[Mesh] OR "Polymyxins/toxicity"[Mesh] ))) OR (( "Colistin/adverse effects"[Mesh] OR "Colistin/therapeutic use"[Mesh] OR "Colistin/toxicity"[Mesh] ))) OR "Polymyxins"[Mesh]) OR Polymyxin) OR colistin) OR "polymyxin b") OR "polymyxin e")) AND (((("Case Reports" [Publication Type]) OR "Clinical Trial" [Publication Type]) OR "Observational Study" [Publication Type]) OR "Comparative Study" [Publication Type]) |
| #4 | Search ((("Case Reports" [Publication Type]) OR "Clinical Trial" [Publication Type]) OR "Observational Study" [Publication Type]) OR "Comparative Study" [Publication Type] |
| #3 | Search (((((((( "Polymyxins/adverse effects"[Mesh] OR "Polymyxins/therapeutic use"[Mesh] OR "Polymyxins/toxicity"[Mesh] ))) OR (( "Colistin/adverse effects"[Mesh] OR "Colistin/therapeutic use"[Mesh] OR "Colistin/toxicity"[Mesh] ))) OR "Polymyxins"[Mesh]) OR Polymyxin) OR colistin) OR "polymyxin b") OR "polymyxin e" |
| #2 | Search ("Acinetobacter baumannii") OR "Acinetobacter baumannii"[Majr] |
| #1 | Search ("Drug Resistance, Bacterial"[Mesh]) |

**Embase**

| **No.** | **Query** |
| --- | --- |
| #23 | #1 AND #9 AND #14 AND #21 |
| #22 | #9 AND #14 AND #16 AND #21 |
| #21 | #17 OR #18 OR #19 OR #20 |
| #20 | polymyxins |
| #19 | 'colistin'/exp/mj |
| #18 | 'polymyxin b'/exp/mj |
| #17 | 'polymyxin'/exp/mj |
| #16 | 'acinetobacter baumannii'/exp/mj |
| #15 | #1 AND #6 AND #9 AND #14 |
| #14 | #10 OR #11 OR #12 OR #13 |
| #13 | 'retrospective study'/exp |
| #12 | 'prospective study' |
| #11 | 'clinical trial'/exp |
| #10 | 'case control study'/exp |
| #9 | 'therapy'/exp |
| #8 | 'meropenem'/exp |
| #7 | 'imipenem'/exp |
| #6 | #2 OR #3 OR #4 OR #5 |
| #5 | polymyxins |
| #4 | 'colistin'/exp |
| #3 | 'polymyxin'/exp |
| #2 | 'polymyxin b'/exp |
| #1 | 'acinetobacter baumannii'/exp |

**Cochrane library**

| **No.** | **Query** |
| --- | --- |
| #9 | #3 and #5 and #8 |
| #8 | #7 or #8 |
| #7 | polymyxin or polymyxins or colistin or “polymyxin b” or “polymyxin e” |
| #6 | “polymyxins” explode all trees |
| #5 | “acinetobacter baumannii” or #2 |
| #4 | “acinetobacter baumannii” explode all trees |
| #3 | #1 or #2 |
| #2 | “drug resistance” explode all trees |
| #1 | “carbapenems” explode all trees |

**CINHAL**

“polymyxin or colistin” and ““acinetobacter baumannii and drug resistant” or “acinetobacter baumannii and carbapenem” or “carbapenem resistant Acinetobacter baumannii””

**ClinicalTrials.gov**

“polymyxin or colistin” and ““carbapenem and Acinetobacter baumannii” or “acinetobacter baumannii and drug resistant””
