# Supplementary material for: Clinical efficacy and safety of polymyxins based versus non-polymyxins based therapies in the infections caused by carbapenem-resistant Acinetobacter baumannii: a systematic review and meta-analysis
Source: BMC Infect Dis. 2020 Apr 21;20:296. doi: 10.1186/s12879-020-05026-2 (PMC7175513; doi:10.1186/s12879-020-05026-2)
Supplement: Supplementary file 2 — Additional file 2: Table S1. Summary of sensitivity analyses. Table S2. Summary of leave-one-out analysis on primary outcome. [file 12879_2020_5026_MOESM2_ESM.docx]

**Table S1.** Summary of sensitivity analyses

| **Outcome** | **Primary analysis (OR/CI)** | **NS and SS** | **Excluding studies at serious risk of bias (OR/CI)** | **NS and SS** | **Excluding studies published before 2010 (OR/CI)** | **NS and SS** | **Excluding studies with inadequate balance at baseline (OR/CI)** | **NS and SS** | **Excluding studies with small sample size (≤ 50) (OR/CI)** | **NS and SS** |
| --- | --- | --- | --- | --- | --- | --- | --- | --- | --- | --- |
| **1-month mortality** | 0.79  (0.43, 1.44) | 11, 496/556 | 1.78  (0.82, 3.85) | 3, 105/68 | 1.06  (0.65, 1.70) | 9, 385/523 | 0.79  (0.43, 1.47) | 9, 415/304 | 0.90  (0.48, 1.68) | 7, 435/494 |
| **Clinical response** | 1.97  (1.30, 2.99) | 5, 253/196 | NA | NA | 1.78  (1.12, 2.83) | 4, 157/176 | ETP | ETP | 2.23  (1.39, 3.58) | 2, 206/148 |
| **Adverse events** | 4.32  (1.39, 13.48) | 4, 61/62 | NA | NA | 7.91  (2.22, 28.10) | 3,  46/49 | ETP | ETP | NA | NA |

NA, no study is eligible or the number of included study was less than three; NS, number of included study; SS, sample size of polymyxins group/non-polymyxins group; ETP, the result is equal to primary analysis.

**Table S2.** Summary of leave-one-out analysis on primary outcome

| **Study** | **OR** | **Lower bound** | **Upper bound** | **SD** | ***P* value** |
| --- | --- | --- | --- | --- | --- |
| Overall | 0.79 | 0.43 | 1.44 | 8.36 | 0.84 |
| -Trottier 2007 | 1.09 | 0.69 | 1.70 | 7.88 | 0.72 |
| -Betrosian 2008 | 0.92 | 0.56 | 1.51 | 7.76 | 0.74 |
| -Lopez-Cortes 2014 | 0.94 | 0.57 | 1.56 | 7.94 | 0.80 |
| -Ozvatan 2016 | 0.92 | 0.54 | 1.57 | 7.60 | 0.76 |
| -Zalts 2016 | 0.87 | 0.54 | 1.39 | 6.70 | 0.55 |
| -Pan 2018 | 1.10 | 0.76 | 1.60 | 6.75 | 0.62 |
| -Khalili 2018 | 0.93 | 0.56 | 1.56 | 8.09 | 0.79 |
| -Liang 2018 | 0.96 | 0.55 | 1.69 | 8.30 | 0.90 |
| -Sipahi 2018 | 0.95 | 0.57 | 1.56 | 8.11 | 0.83 |
| -Chusri 2019 | 0.97 | 0.58 | 1.59 | 8.24 | 0.89 |
| -Raz-Pasteur 2019 | 0.81 | 0.47 | 1.39 | 7.10 | 0.44 |
