# Supplementary material for: Clinical efficacy and safety of polymyxins based versus non-polymyxins based therapies in the infections caused by carbapenem-resistant Acinetobacter baumannii: a systematic review and meta-analysis
Source: BMC Infect Dis. 2020 Apr 21;20:296. doi: 10.1186/s12879-020-05026-2 (PMC7175513; doi:10.1186/s12879-020-05026-2)
Supplement: Supplementary file 3 — Additional file 3. Funnel plot showing publication bias of 1-month mortality. [file 12879_2020_5026_MOESM3_ESM.pdf]

Additional file 3

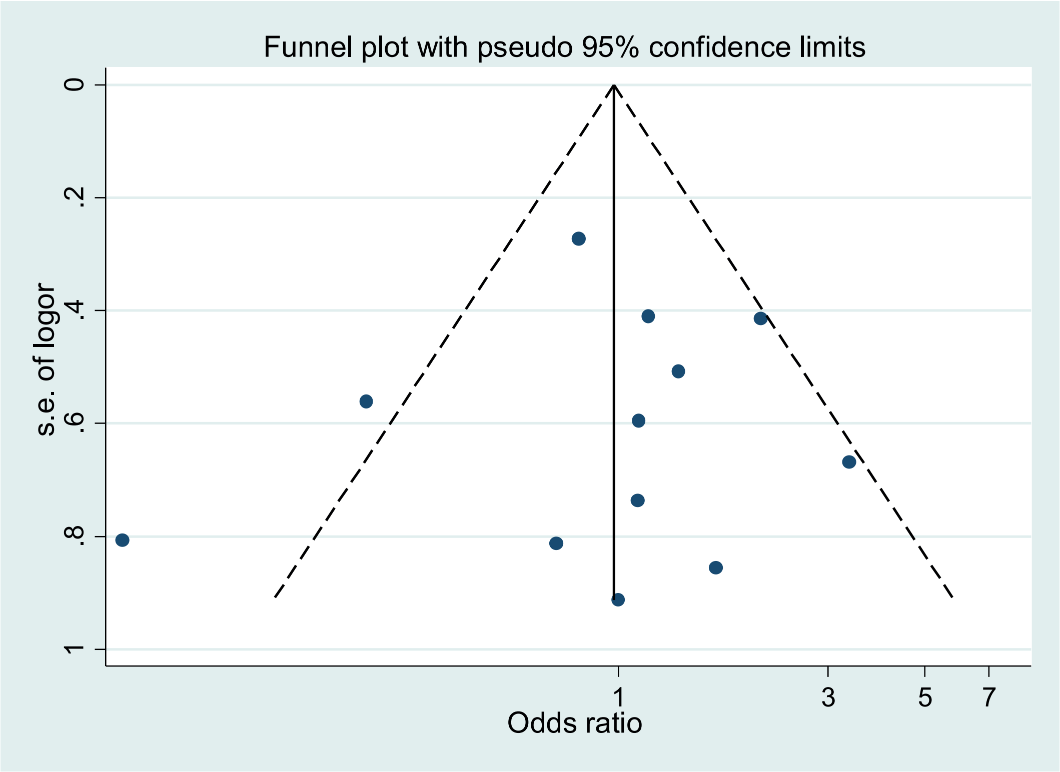

Funnel plot showing publication bias of 1-month mortality. Each point represents the odds ratio (OR) and the standard error of logOR for a single study.
